# Identification and functional characterization of mRNAs that exhibit stop codon readthrough in *Arabidopsis thaliana*

**DOI:** 10.1016/j.jbc.2022.102173

**Published:** 2022-06-22

**Authors:** Sarthak Sahoo, Divyoj Singh, Anumeha Singh, Madhuparna Pandit, Kirtana Vasu, Saubhik Som, Naga Jyothi Pullagurla, Debabrata Laha, Sandeep M. Eswarappa

**Affiliations:** 1 Undergraduate Program, Indian Institute of Science, Bengaluru, India; 2 Department of Biochemistry, Indian Institute of Science, Bengaluru, India

**Keywords:** stop codon, readthrough, ribosomes, translation, Arabidopsis, CDS, coding sequence, IDR, intrinsically disordered region, ISR, interstop codon region, NLS, nuclear localization signal, PTS, peroxisomal targeting sequence, SCR, stop codon readthrough, SRA, Sequence Read Archive

## Abstract

Stop codon readthrough (SCR) is the process of continuation of translation beyond the stop codon, generating protein isoforms with C-terminal extensions. SCR has been observed in viruses, fungi, and multicellular organisms, including mammals. However, SCR is largely unexplored in plants. In this study, we have analyzed ribosome profiling datasets to identify mRNAs that exhibit SCR in *Arabidopsis thaliana*. Analyses of the ribosome density, ribosome coverage, and three-nucleotide periodicity of the ribosome profiling reads in the mRNA region downstream of the stop codon provided strong evidence for SCR in mRNAs of 144 genes. We show that SCR generated putative evolutionarily conserved nuclear localization signals, transmembrane helices, and intrinsically disordered regions in the C-terminal extensions of several of these proteins. Furthermore, gene ontology functional enrichment analysis revealed that these 144 genes belong to three major functional groups—translation, photosynthesis, and abiotic stress tolerance. Using a luminescence-based readthrough assay, we experimentally demonstrated SCR in representative mRNAs belonging to each of these functional classes. Finally, using microscopy, we show that the SCR product of one gene that contains a nuclear localization signal at the C-terminal extension, *CURT1B*, localizes to the nucleus as predicted. Based on these observations, we propose that SCR plays an important role in plant physiology by regulating protein localization and function.

A stop codon on an mRNA signals the translating ribosomes to terminate the process of translation. However, in certain mRNAs, ribosomes fail to terminate at the canonical stop codon and continue translation till the next in-frame stop codon. This is caused by recoding of stop codons by a near-cognate tRNA or a suppressor tRNA. This process of stop codon readthrough (SCR) generates protein isoforms with extended C terminus, thus contributing to proteome expansion (1, 2). Because of the extended C terminus, the protein isoform generated by SCR can be different from the canonical isoform in terms of its localization, function, or stability (3, 4, 5, 6). Since this process occurs at the translational level, SCR enables cells to swiftly respond to environmental cues.

SCR has been observed in bacteria, yeast, insects, mammals, and viruses. About 5% of *Saccharomyces cerevisiae* genes are targets of SCR (7). mRNAs of 283 *Drosophila* genes exhibit signs of SCR (8). In cases of mammals, SCR has been demonstrated only in 14 mammalian mRNAs. SCR is usually regulated by *cis*-acting RNA element (*e.g.*, pseudoknot and stem-loop structures) present downstream of the stop codons (9). *trans*-acting factors (RNA-binding proteins or microRNAs) also regulate SCR in certain cases (2, 3, 5). These factors possibly cause ribosomal pausing at the stop codon, which provides sufficient opportunity for the near-cognate tRNAs to recognize the stop codon, resulting in SCR (2). The context of the stop codon can make an mRNA permissive for SCR. For example, the sequence CUAG immediately after the stop codon is critical for the SCR in some mammalian mRNAs (*e.g.*, *OPRL1*) (10).

The SCR products can have properties different from those of the canonical isoforms due to the C-terminal extension. For example, they can have different localization (*e.g.*, *LDHB* and *MDH1*) (6, 11, 12), attenuated functions (*e.g.*, *VEGFA*) (3), different functions (*e.g.*, *AGO1*) (13), and reduced stability (*e.g.*, *MTCH2*) (4). Multiple physiological processes are regulated by SCR, for example, tracheal development in *Drosophila* (14), angiogenesis (3), microRNA pathway (5), mitochondrial function (4), myelination (15), and replication of retroviruses (16).

SCR is well-studied in plant viruses (17, 18). For example, tobacco necrosis virus-D expresses its polymerase, and potato leafroll virus generates a minor capsid protein by SCR (19, 20). It enables viruses to maximize the coding potential of their compact genome. Since plant viruses utilize the translation machinery of the host, it is likely that some plant mRNAs are subjected to SCR. However, so far, there is only one report of SCR in a plant mRNA. SCR in *Arabidopsis* eRF1-1 mRNA regulates its expression by protecting the mRNA from nonsense–mediated decay (21). A wide range of other translation regulation mechanisms have been observed during plant development, light-dark cycle, viral infections, and environmental stresses (22). A genome-wide analysis of SCR, which is also a translation regulation mechanism, is lacking to understand its role in plant physiology.

Ribosome profiling technique, based on the deep sequencing of ribosome protected RNA fragments, has revolutionized our understanding of the process of translation and its regulation. Because it reveals ribosome-occupied regions on an mRNA, ribosome profiling has the potential to identify novel translation events such as SCR (23). In this study, we analyzed ribosome profiling datasets and identified 144 genes of *Arabidopsis thaliana* as targets of SCR. Further, we experimentally confirmed this process in five candidate genes.

## Results

### Selection and curation of ribosome profiling datasets

The presence of translating ribosomes after the canonical stop codon of an mRNA strongly indicates SCR (23). We analyzed ribosome profiling data generated from *A. thaliana*, which are available at Sequence Read Archive (SRA) of National Center for Biotechnology Information. We retrieved 14 *A. thaliana* ribosome profiling datasets from SRA and processed them as described in Experimental procedures.

**Figure 1:**
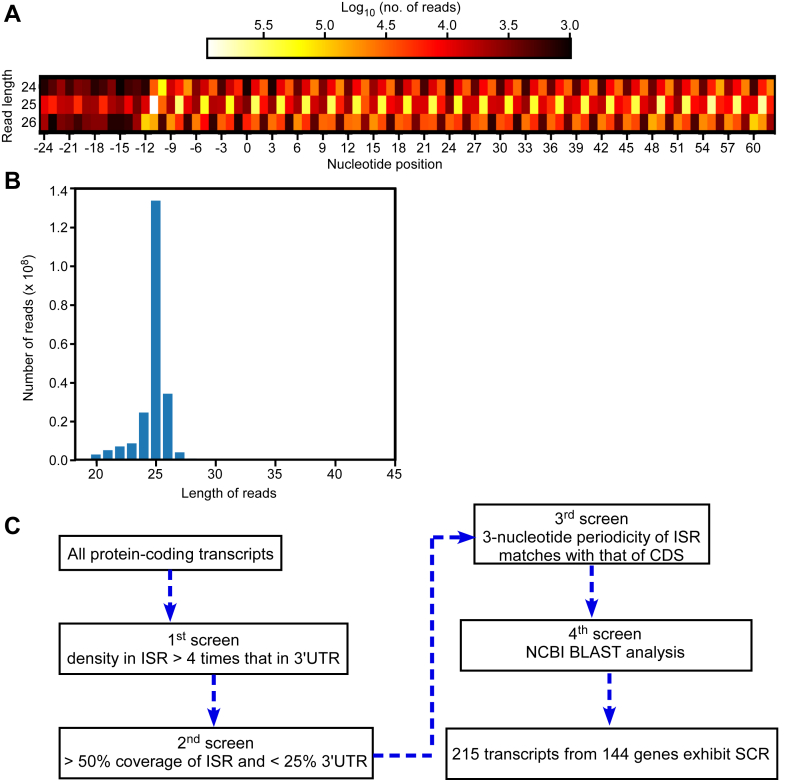
**Selection and analysis of ribosome profiling datasets.***A*, heat map showing the three-nucleotide periodicity profile of the dataset SRP074840. Ribosomal footprints on all coding sequences were analyzed to get this profile for read lengths 24, 25, and 26. Reads were assigned to three frames based on which frame the first nucleotide aligns with on an mRNA sequence. The start codon ATG is indicated by the position 0 on the X-axis. *B*, distribution of ribo-seq read lengths. The graph shown is from the dataset SRP074840. *C*, flow chart showing the four-level screening method to identify mRNAs that show SCR in *A. thaliana*. Another flow chart with more details is shown in Fig. S2. CDS, coding sequence; ISR, interstop codon region; SCR, stop codon readthrough; UTR, untranslated region.

Ribosomal footprints obtained from translating ribosomes exhibit frame bias, *i.e.*, they show a fixed distribution of reads across the three frames of the coding sequence (CDS). This three-nucleotide periodicity (or phasing) is a sign of translation on the corresponding region of an mRNA (in our case, the proximal 3′UTR [untranslated region]). This kind of spatial resolution along mRNAs is required in ribosome profiling datasets to claim unusual translation events such as SCR. Therefore, we first analyzed the three-nucleotide periodicity profile of the ribosome profiling datasets. We chose nine ribosome profiling datasets based on a clear three-nucleotide periodicity of ribosome profiling (ribo-seq) reads corresponding to the CDSs of all genes. A representative profile is shown in Figure 1*A*.

These nine datasets were derived from seedlings, root, shoot, flower, and from a cell line of *A. thaliana* (Table S1) (24, 25, 26, 27, 28, 29, 30, 31). We then analyzed the length distribution of the reads in each dataset. Length distribution was consistent with footprints of 80S ribosomes, which is a signature of translation. A representative length distribution is shown in Figure 1*B*. Based on this distribution, we chose the reads of three most abundant lengths for further analyses.

After removing the reads that map onto noncoding RNAs, we aligned the rest of the reads with *A. thaliana* protein-coding mRNAs. Only those reads that align 100% (*i.e.*, without any mismatch) with an mRNA were considered for the analysis. As our aim was to identify SCR events, we focused on the ribosome footprints in the proximal part of the 3′UTR—from the canonical stop codon to the downstream in-frame stop codon (Fig. S1). This region was termed the interstop codon region (ISR). mRNAs without downstream in-frame stop codon were not included in the analysis.

### Two hundred fifteen mRNAs from 144 genes of *A. thaliana* show evidence of SCR

We subjected the mRNAs of *A. thaliana* to a stringent four-level screening to identify the targets of SCR (Figs. 1*C* and S2). It is important to distinguish ribosome profiling (ribo-seq) reads due to SCR from reads resulting from nontranslating events (32). This was achieved by comparing the ribosome densities in different regions of an mRNA. The average ribosome density in the 3′UTR of mRNAs indicates ribosome occupancy due to events not related to translation. mRNAs that showed at least 4-fold higher ribosome density in the ISR compared to the rest of the 3′UTR were considered for further analysis. One thousand one hundred forty-four mRNAs were identified in this first level of screening (Fig. S2 and Table S1).

It is possible that the increased ribosome density can be due to a specific segment of the ISR with a strong RNA structure or a strong interaction with a protein (or any *trans*-acting molecule). To exclude such events, we subjected the mRNAs to a coverage-based filtering. We eliminated mRNAs with <50% coverage in the ISR and >25% coverage in the rest of the 3′UTR. Five hundred fifty mRNAs satisfied this criterion, and all of them had at least one ribo-seq read spanning the canonical stop codon (Fig. S2 and Table S1).

**Figure 2:**
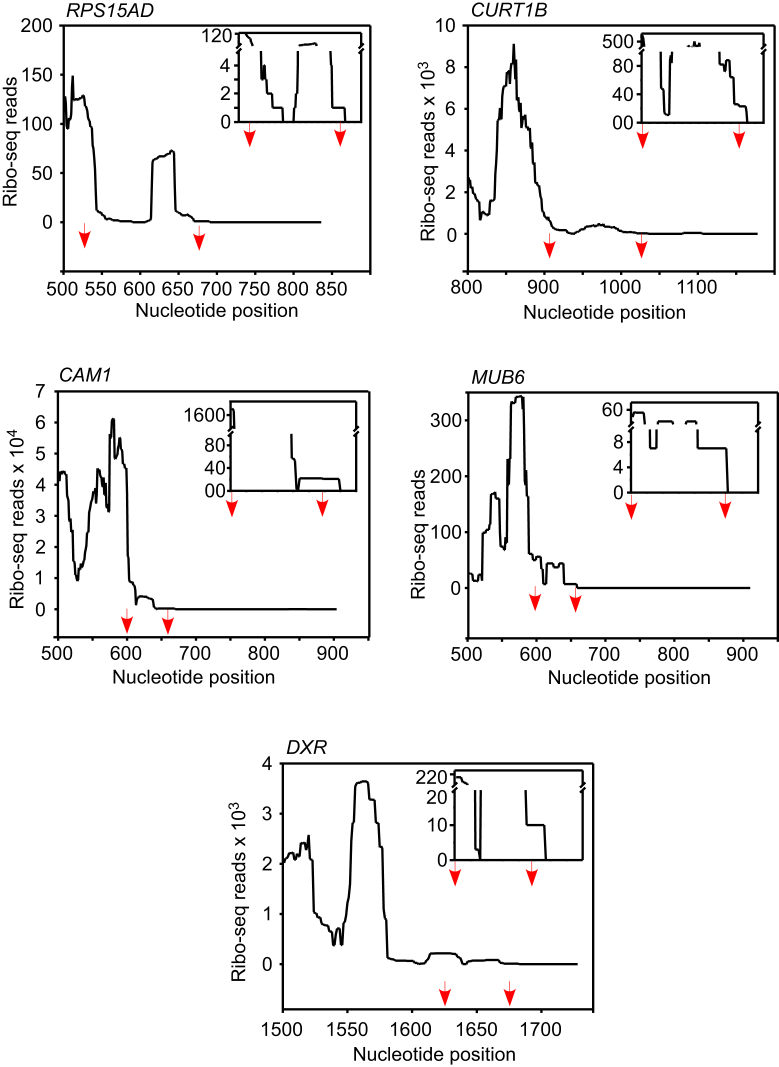
**Ribosomal density in the ISR of five SCR-positive mRNAs—*RPS15AD*, *CURT1B*, *CAM1*, *MUB6*, and *DXR*.** Graphs show ribo-seq reads in the ISR. *Red arrows* indicate the position of the two stop codons. Adjacent parts of the coding sequence and the 3′UTR are also shown for comparison. The data shown are from the dataset SRP074840. Inset show zoomed-in graphs. CDS, coding sequence; ISR, interstop codon region; SCR, stop codon readthrough; UTR, untranslated region.

**Figure 3:**
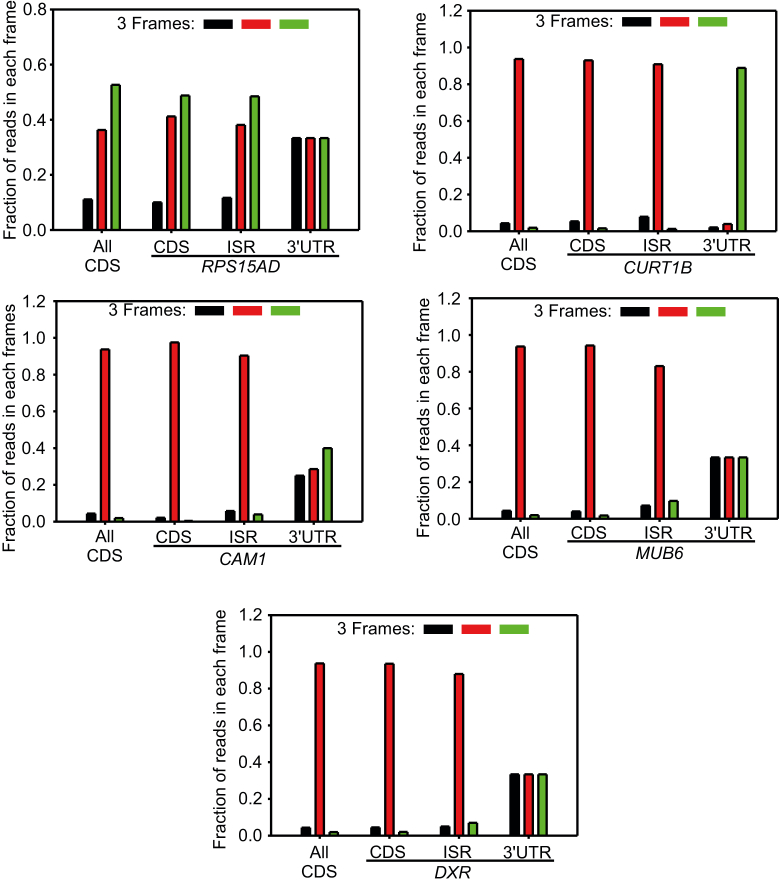
**Three-nucleotide periodicity at the ISR of five SCR-positive mRNAs—*RPS15AD*, *CURT1B*, *CAM1*, *MUB6*, and *DXR*.** Graphs show fraction of ribo-seq reads in three translation frames. The three-nucleotide periodicity profile of coding sequences of all protein-coding genes is shown for comparison (All CDS; see Fig. S3). Graphs are derived from the dataset SRP160376 (for *RPS15AD*) and SRP074840 (for others). CDS, coding sequence; ISR, interstop codon region; SCR, stop codon readthrough; UTR, untranslated region.

The three-nucleotide periodicity profile of the ribo-seq reads assigned to ISR, similar to that of the reads assigned to the CDS, provides strong evidence for SCR. Two hundred thirty-six mRNAs satisfied this third level of screening. It is possible that the remaining mRNAs may include ribosomal frameshifting (also known as translational frameshifting) candidates. We did not pursue frameshifting events as the focus of our study was SCR, where the frame of the ribosomes translating the ISR is same as that in the CDS. The ribosome density profile and the three-nucleotide periodicity profile of the ISRs of five representative genes—*RPS15AD*, *CURT1B*, *CAM1*, *DXR*, and *MUB6*—are shown in Figures 2 and 3 and Fig. S3.

The mRNA sequences of *A. thaliana* were retrieved from Ensembl Plants. The annotation of the CDS on mRNAs can vary across the databases, providing false-positive evidence for SCR. To rule this out, we performed BLAST analysis for the peptides encoded by the ISRs of 236 mRNAs that passed the screening described above, against National Center for Biotechnology Information’s protein database for *A. thaliana*. We found 21 matches, which were eliminated in this fourth level screening.

**Figure 4:**
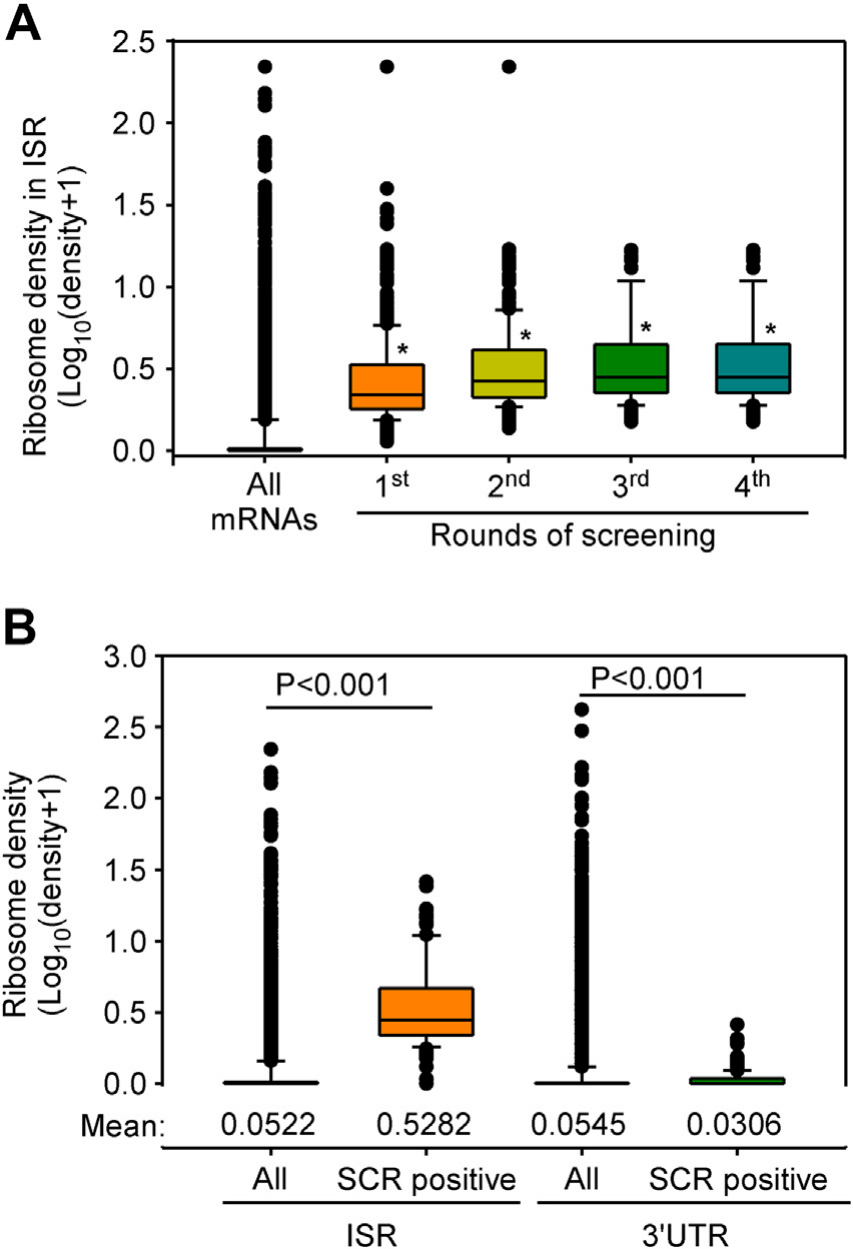
**Ribosomal density in the ISR of SCR-positive mRNAs.***A*, the graph shows the increase in ribosome density in the ISRs after each round of screening. ∗*p* < 0.001 Mann–Whitney rank sum test (compared to ‘All mRNAs’). *B*, graph shows the comparison of ribosomal density in the ISR *versus* that in the 3′UTR. This comparison is shown for SCR-positive mRNAs and for all mRNAs. *p* values were calculated using Mann–Whitney rank sum test. *Numbers* at the *bottom* of the graph indicate the mean value. The *box* represents 25% and 75% values, and the *horizontal line* within the *box* shows the median value. The analysis shown is for the dataset SRP074840. ISR, interstop codon region; SCR, stop codon readthrough; UTR, untranslated region.

Thus, 215 mRNAs encoded by 144 genes of *A. thaliana* passed our stringent four-level screening, and they were designated as SCR-positive genes (Fig. S2 and Table S2). The average ribosome density in the ISR increased with each screening step (Fig. 4*A*). Also, the average ribosome density in the ISR of SCR-positive mRNAs was 10-fold higher compared to the same in all mRNAs. This difference was not observed in the ribosome density of the 3′UTR excluding the ISR (Fig. 4*B*).

These results show that our screening methods were able to identify translational events immediately after the stop codon, which constitutes SCR. Though translational frameshifting could also result in increased ribosomal density in the 3′UTR, the three-nucleotide periodicity-based third screen will remove frameshifting events as described above. We have not allowed a single mismatch while assigning reads to different regions of an mRNA. Also, all SCR-positive candidates have at least one ribosome profiling read mapping on to the region spanning the canonical stop codon (the junction of the CDS and the ISR). These two conditions rule out RNA editing and polymorphism at the stop codon as reasons for ribosome footprints after the stop codon.

In our analyses, we have excluded genes which have ISRs and/or 3′UTRs <45 nucleotides and genes with <30 reads in their ISR. Also, our method does not reveal SCR events that occur under specific physiological or pathological conditions not included in the ribosome profiling studies. Hence, 144 SCR-positive genes is an underestimate; it is likely that more mRNAs exhibit SCR in *A. thaliana*. For example, we did not observe any ribo-seq footprints in the ISR of *eRF1-1*, whose SCR has been demonstrated in *A. thaliana* (21).

### The stop codon TGA is enriched in SCR-positive genes

**Figure 5:**
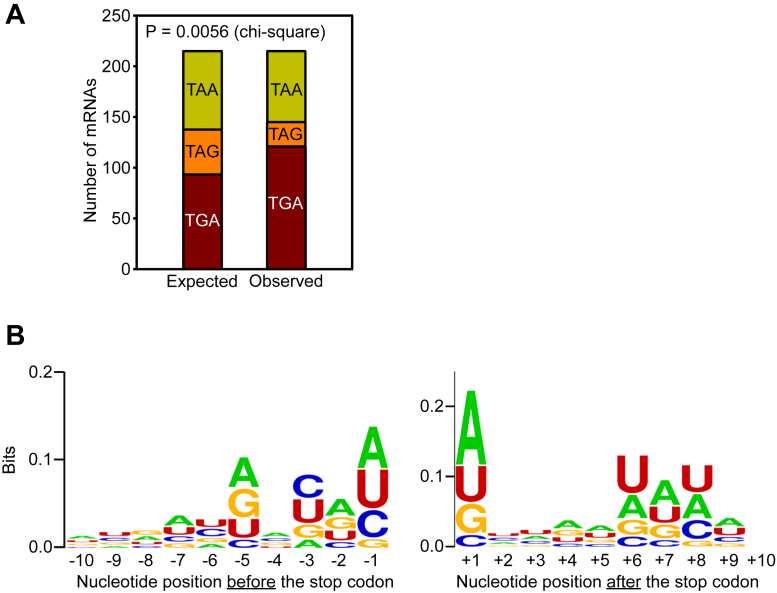
**The canonical stop codon and its context in SCR-positive mRNAs.***A*, distribution of the three stop codons in SCR-positive mRNAs. Expected values were obtained based on their occurrence in all mRNAs of *A. thaliana*. *B*, sequence logo of the stop codon context of SCR-positive mRNAs. The analysis was performed using WebLogo. SCR, stop codon readthrough.

Since the identity of the stop codons can influence the efficiency of translation termination (33), we examined the distribution of the three stop codons among the SCR-positive mRNAs at their canonical termination position. We observed a 25% higher occurrence of TGA stop codon in SCR-positive mRNAs compared to the expected frequency (Fig. 5*A*). Interestingly, TGA is the leakiest among the three stop codons, which facilitates the process of SCR.

The context of stop codons, especially the nucleotides immediately before (−1) and after (+1) the stop codon, can also influence the efficiency of translation termination (34). Hence, we examined if there are any conserved sequences around the stop codon in SCR-positive mRNAs. We used WebLogo, a sequence logo generator, to visualize the extent of conservation around the stop codon (35). Here, the height of the stack indicates the extent of conservation at that particular position. Interestingly, nucleotides just before (−1) and after (+1) and the stop codon showed higher conservation compared to other positions. A and U were more frequently observed in these positions than the other two nucleotides (Fig. 5*B*). These conserved residues are possibly important to provide SCR-permissive context in SCR-positive mRNAs.

### Gene ontology analysis: Genes involved in translation, photosynthesis, and stress response are enriched in SCR-positive genes

**Figure 6:**
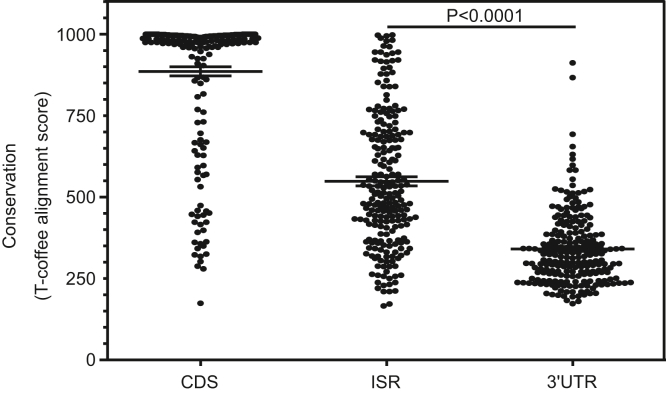
**Evolutionary conservation of the ISRs of SCR-positive genes.** Amino acid sequences encoded by the CDS, ISR, and 3′UTR (excluding the ISR) of SCR-positive genes belonging to the plant species of Brassicacae family were aligned, and the alignment score was calculated using T-Coffee multiple sequence alignment program. Student’s *t* test with Welch’s correction was used to calculate the *p* value. *Horizontal lines* represent mean with 95% confidence interval. CDS, coding sequence; ISR, interstop codon region; SCR, stop codon readthrough; UTR, untranslated region.

Evolutionary conservation at the amino acid level is an indication of functional significance of that region in protein-coding genes. We analyzed the evolutionary conservation of amino acid sequences potentially encoded by the ISRs of all SCR-positive genes, across seven plant species belonging to Brassicaceae family (*A. thaliana*, *Arabidopsis lyrata*, *Brassica rapa*, *Camelina sativa*, *Capsella rubella*, *Eutrema salsugineum*, *Raphanus sativus*). The average amino acid conservation at the ISR was significantly higher compared to that in rest of the 3′UTR, indicating functional significance of SCR (Fig. 6). Absence of conservation in some SCR-positive genes could imply that those SCR events have appeared later during evolution and therefore are specific to *A thaliana*.

**Figure 7:**
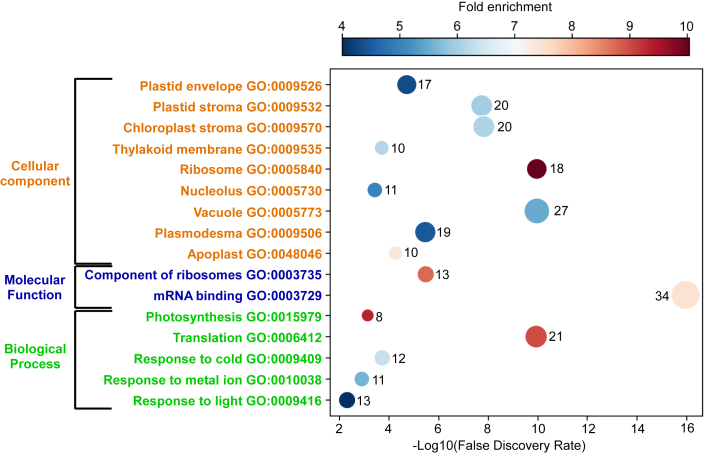
**Gene ontology analysis of SCR-positive genes of *A. thaliana*.** Results of gene ontology (GO) functional enrichment analysis on SCR-positive genes using PANTHER web server. The X-axis shows false discovery rate, and the Y-axis shows multiple functional classes enriched in SCR-positive genes. Color of the *circle* indicates fold enrichment. The number of SCR-positive genes showing enrichment in a functional group is shown next to the *circle*. Size of the *circle* is proportional to this number. SCR-positive genes with more than 4-fold enrichment are shown here. SCR, stop codon readthrough.

We performed gene ontology functional enrichment analysis on 144 SCR-positive genes using PANTHER web server (36). Among biological processes, we observed that genes involved in translation, photosynthesis, and abiotic stress response were enriched in the list of SCR-positive genes. For instance, 21 genes involved in translation were part of this list. In consistence with this, genes encoding proteins localized in ribosomes and nucleolus (site of ribosome assembly) were enriched. With respect to molecular functions, there was an enrichment of genes encoding components of ribosomes and mRNA-binding proteins. Interestingly, 34 out of 144 SCR-positive genes encode RNA-binding proteins. This is much higher than expected as only ∼4% of the protein-coding genes encode RNA binding proteins in *A. thaliana* (37). Proteins encoded by 20 of them localize in chloroplast, and 18 of them are ribosomal proteins (Fig. 7). Together, these observations indicate that SCR could play an important role in regulating the process of translation, photosynthesis, and abiotic stress response in *A. thaliana*.

### SCR can change the localization of the proteins

SCR has been shown to change the localization of the protein product in some cases. For example, the double SCR product of mammalian *MTCH2* is localized to the cytoplasm while the canonical isoform is a mitochondrial membrane protein (4). SCR products of mammalian malate dehydrogenase and lactate dehydrogenase have a peroxisomal targeting sequence (PTS) at the C terminus, which directs them to peroxisomes. However, the canonical isoforms are found in the cytoplasm or mitochondria (6, 11, 12).

We investigated the C-terminal extensions of products of 144 SCR-positive genes for potential signals or motifs that can change the localization of the SCR isoform. Using available computational tools, we searched for nuclear localization signal (NLS), PTS, transmembrane helix, intrinsically disordered region (IDR), isoprenylation sites, and endoplasmic reticulum retention signal (KDEL). We considered only those signals/motifs which are evolutionarily conserved in *A. thaliana* and at least two other plant species not belonging to the genus *Arabidopsis*.

NLS at the C terminus of the SCR products can drive them to the nucleus. We searched for the presence of NLS in the ISR of the 144 SCR products using two different tools, NLStradamus and LOCALIZER (38, 39). Three of them showed NLS in both tools—*CURT1B*, *KCS12*, and *At5g56200*. Also, the putative NLS encoded by the ISR of these three genes were evolutionarily conserved underscoring their functional significance (Fig. S4). Among the three, the canonical protein isoforms of *CURT1B* (The P subunit of Photosystem I) and *KCS12* (3-ketoacyl-CoA synthase) are not localized in the nucleus. Our analysis predicts that their SCR isoforms are localized in the nucleus, possibly with a moonlighting function (Table 1).

**Table 1:** 

We then searched for a putative transmembrane helix at the C terminus of 144 SCR isoforms using TMHMM Server v. 2.0 (40), as this can be a mechanism to change the localization of the protein to a membrane. Eight of the SCR products showed a transmembrane helix at their extended C terminus (Table 2). These putative transmembrane helices were observed in the corresponding ISRs of at least two other species belonging to Brassicaceae family (Figs. S5 and S6). The canonical isoforms of six of these eight genes are not known to be membrane proteins. Thus, SCR can potentially regulate their function by driving them to the cell or organelle membrane, as they are exposed to new microenvironment and interacting proteins near the membrane (41).

**Table 2:** 

In case of mammalian *VEGFA* and *AGO1*, SCR results in a C terminus with predicted IDR. This changes the functional properties of their SCR isoforms (3, 5). We analyzed the products of 144 SCR-positive genes for possible IDRs at the C terminus using the IUPred2A tool (42). We observed IDR in the C terminus of the SCR products of four genes. These putative IDRs were observed in the corresponding ISRs of at least two other species belonging to Brassicaceae family (Fig. S7). They are involved in the organization of cytoskeleton, redox homeostasis, fatty acid synthesis, auxin, and hypersensitive response (Table 3). IDRs in their C termini can potentially alter the functional properties of these four SCR products.

**Table 3:** 

We did not observe evolutionarily conserved PTS, isoprenylation sites, and endoplasmic reticulum retention signal at the C terminus of any of the SCR-positive genes.

### Experimental validation of SCR

We performed *in vitro* translation experiments using wheat germ extract to validate SCR in five genes: *RPS15AD* encodes a ribosomal protein; *CURT1B* encodes the P subunit of Photosystem I; *CAM1* encodes calmodulin, which is involved in abiotic stress response (43). These three genes represent three functional classes that are enriched in SCR-positive genes—translation, photosynthesis, and abiotic stress response. We also selected *MUB6* (encodes membrane-anchored ubiquitin-fold protein 6) and *DXR* (encodes a chloroplast enzyme required for isoprenoid biosynthesis), which do not belong to any of these three classes but are SCR-positive. As described above, ribosome footprints were observed after the stop codon in the ISR of these mRNAs (Fig. 2). Also, the three-nucleotide periodicity of the ribosomal footprints on the ISR was comparable to that of the CDS but not to that of the 3′UTR (Fig. 3). We used *ADF1* (encodes an actin depolymerizing factor) and *UPL7* (encodes a ubiquitin protein ligase) as negative controls in *in vitro* SCR assays as these two genes were eliminated in the first round of our screening.

**Figure 8:**
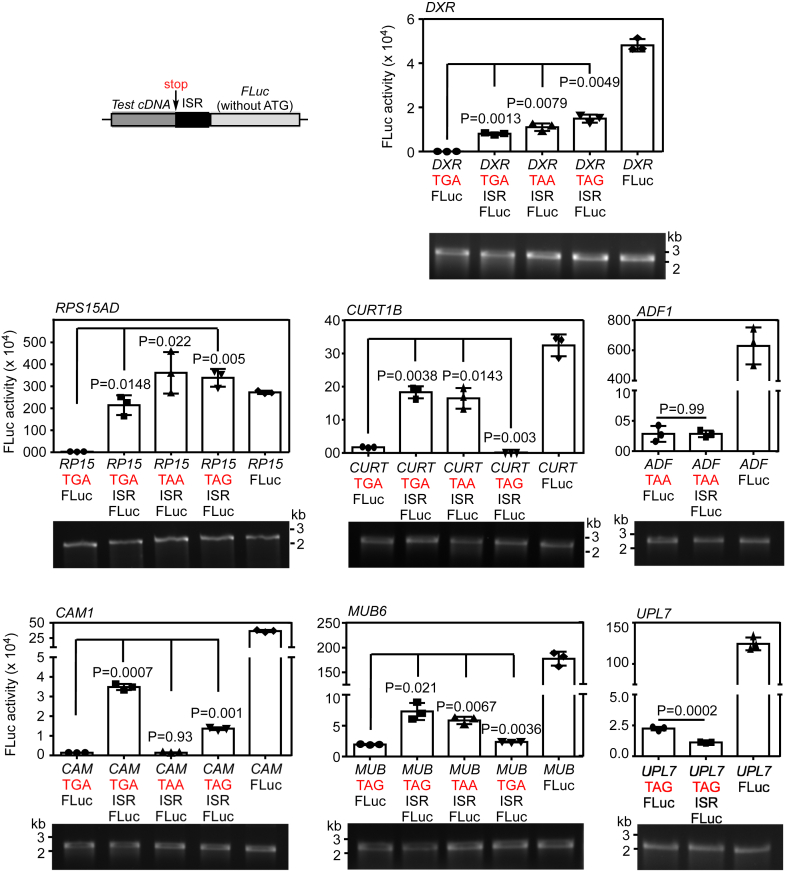
**Experimental validation of SCR in five *A. thaliana* mRNAs—*DXR*, *RPS15AD*, *CURT1B*, *CAM1*, and *MUB6*.** Luminescence-based SCR assay. cDNA of a test gene along with the ISR was cloned upstream of and in-frame with the cDNA of firefly luciferase (FLuc) such that FLuc is expressed only if there is SCR across the stop codon of the test cDNA (see the schematic). *ADF1* and *UPL7* were used as negative controls. Constructs were subjected to *in vitro* transcription followed by *in vitro* translation using wheat germ extract with equal amount of RNA. Expression of Fluc was measured by its luminescence activity, which is shown in the graphs. Constructs without ISR were used to measure background signal (*first bar*), and constructs without any stop codon between the test cDNA and the FLuc were used to measure the maximum luminescence activity (*last bar*). *Second bar* in all graphs represents SCR activity across the canonical stop codon. Activity across mutated stop codons are also shown. Statistical significance was calculated using Student’s *t* test. *p*-values (two-sided) shown are in comparison with the constructs without the ISR. Welch’s correction was applied wherever required. *Bars* show mean ± SD (n = 3). Input RNA obtained by *in vitro* transcription is shown below the graphs. Y-axis of all graphs shows FLuc activity (×10^4^). ISR, interstop codon region; SCR, stop codon readthrough.

Luminescence-based SCR assays were performed as described previously (5). We cloned the cDNAs of these genes upstream of and in-frame with the cDNA of firefly luciferase without its start codon. Constructs without the corresponding ISRs were used to know the background level of luciferase activity. Luminescence will be observed only if translation continues across the canonical stop codon of the test cDNA (Schematic in Fig. 8). Thus, luminescence in these assays indicates SCR. *In vitro* transcription followed by *in vitro* translation using wheat germ extract revealed significant luminescence activity in mRNAs of all five SCR-positive genes, much above the background level. However, this was not observed in *ADF1* and *UPL7*, two negative controls without significant ribosome density in their ISRs. A construct without a stop codon between the test cDNA and the firefly luciferase cDNA was used to measure the efficiency of SCR. This analysis revealed 17%, 79%, 56%, 10%, and 4% SCR in *DXR*, *RPS15AD*, *CURT1B*, *CAM1*, and *MUB6*, respectively. The two negative controls, *ADF1* and *UPL7*, showed less than 1% SCR, which was comparable to background level (Fig. 8). Further, SCR of these five genes was observed in a mammalian *in vitro* translation system based on rabbit reticulocyte lysate (Fig. S8). Together, these results demonstrate SCR of these five genes and validate our ribosome-profiling–based screening method.

To understand the importance of the identity of the stop codons in SCR, we mutated the canonical stop codon of these five genes to other two stop codons and performed *in vitro* SCR assay. In case of *CAM1* and *CURT1B*, we observed inhibition of SCR in one of the stop codon mutations. In other candidates tested, SCR was observed across all three stop codons though at varying efficiency. Stop codon identity–dependent and –independent SCRs have been reported in other systems also. For example, SCR in *VEGFA* does not depend on the identity of the stop codon (3). However, SCR in mammalian *AGO1* and tobacco necrosis virus-D depends on it (5, 20).

### SCR product of CURT1B localizes to the nucleus

**Figure 9:**
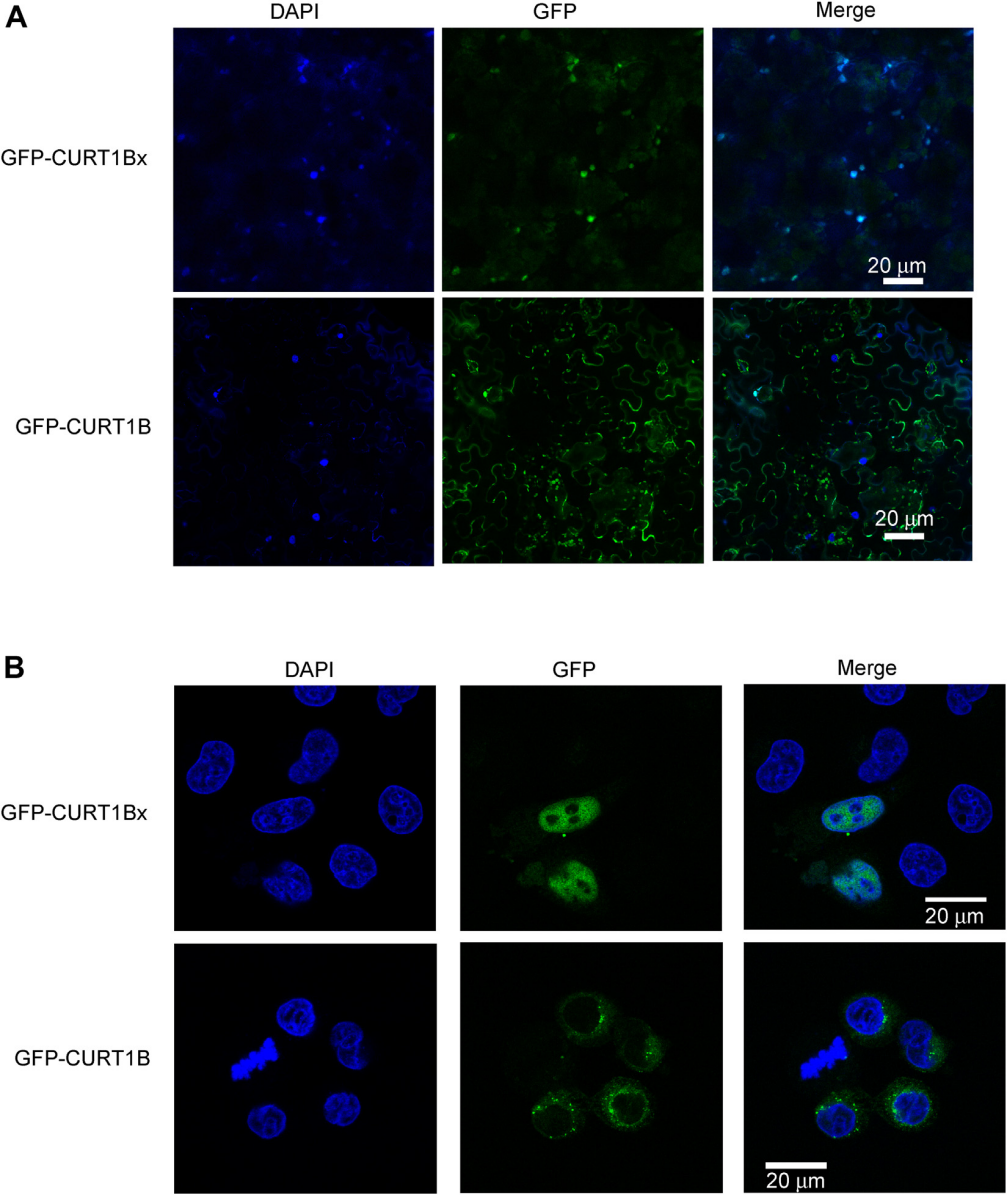
**Nuclear localization of the SCR product of CURT1B (CURT1Bx).***A*, confocal microscopy images of *N. benthamiana* leaves expressing G3GFP-CURT1B or G3GFP-CURT1Bx showing their localization with respect to the nucleus (DAPI). Leaves were infiltrated with *Agrobacterium* harboring G3GFP-CURT1B or G3GFP-CURT1Bx constructs. Brightness and contrast were increased equally across the two channels. *B*, HeLa cells expressing GFP-CURT1B or GFP-CURT1Bx showing their localization with respect to the nucleus (DAPI). Cells were transfected with plasmids expressing GFP-CURT1B or GFP-CURT1Bx. Images are representatives from two independent experiments. SCR, stop codon readthrough.

Finally, we experimentally tested the predicted nuclear localization of the SCR product of *CURT1B* (termed CURT1Bx). For this, we expressed G3GFP-CURT1B or G3GFP-CURT1Bx in *Nicotiana benthamiana* leaves using *Agrobacterium*-mediated plant transformation method and performed confocal microscopy to image the localization of G3GFP-tagged proteins. *N. benthamiana* was chosen as its leaves are amenable for *Agrobacterium* infiltration and expression of exogenous cDNAs. As predicted, we observed that CURT1Bx is localized in the DAPI-stained nuclei. However, the CURT1B did not show nuclear localization (Fig. 9*A*). Since NLSs are conserved in eukaryotes, we tested the localization of CURT1Bx in HeLa cells (mammalian cell) also. Similar to *N. benthamiana* leaves, we observed nuclear localization of CURT1Bx, but not CURT1B (Fig. 9*B*).

## Discussion

Our analysis of ribosome profiling datasets provides strong evidence for SCR in mRNAs of 144 genes of *A. thaliana*. Using a similar analysis of ribosome profiling data, mRNAs of 350 *Drosophila* genes and 42 human genes have been predicted to exhibit SCR (23). The advent of ribosome profiling technique has revealed previously unknown (or lesser known) mechanisms of translational regulation, including SCR. Since this technique is based on experimentally generated ribosome footprints on mRNAs, it is superior to evolutionary conservation-based computational screening methods to detect SCR, which will miss SCR events that have emerged relatively recently during evolution. Furthermore, the nucleotide resolution of ribosome profiling enables us to decipher the frame of translation at the ISRs. The distribution of length of ribosome profiling reads will have a signature of 80S ribosome occupancy. These features are important to distinguish SCR from ribosomal frameshifting and nontranslational events (*e.g.*, protein binding and RNA structures) (44). Thus, ribosome profiling is a powerful tool to identify SCR events.

It would be remarkable if SCR does change the properties of the proteins in multiple ways as predicted by our analyses and as demonstrated in case of *CURT1B*—by introducing NLS, transmembrane helices, and IDR in the ISR-encoded C-terminal extension. Though we observed putative PTS in four SCR-positive genes, they were not evolutionarily conserved. Other mechanisms such as posttranslational modification and degradation, which cannot be predicted with high confidence, might also occur at ISR-encoded extensions. This is not very surprising as random peptide sequences have been shown to have functional motifs. For example, 1/5th of randomly generated peptide sequences carry export signal in *S. cerevisiae* (45). Also, in another study involving *S. cerevisiae*, eight out of 28 randomly generated peptides showed multiple organellar localization signals (46). Therefore, for a gene, the chances of acquiring novel functions by SCR are high.

As shown in mammalian and viral SCR processes, it is likely that the nucleotide sequence of ISR is responsible for driving the SCR *via* a *cis*-acting RNA motif or *trans*-acting molecule (3, 5, 9, 47). Thus, ISR likely possesses a dual function—driving the SCR and altering the properties of the SCR product. It will be interesting to study how natural selection will shape such genomic regions with constraints at both nucleotide (ability to induce SCR) and amino acid level (novel function). Another possibility is that the CDS region just upstream of the stop codon of an SCR-positive gene may also play a role in inducing or regulating the SCR. The amino acid sequences encoded in this region can interact with the components of the exit tunnel of ribosomes leading to ribosome stalling, which can provide enough opportunity for the near-cognate tRNAs or suppressor tRNAs to recognize the stop codon as sense codon resulting in SCR (2, 48). Such sequences, termed as ‘ribosome arrest peptides’, regulate translation (*e.g.*, *CGS1* of *A. thaliana*) (49).

The gene ontology analysis suggests that SCR in *A. thaliana* influences three major physiological processes in plants—protein synthesis, photosynthesis, and stress tolerance. Our *in vitro* translation experiments performed using a plant-based system show that the efficiency of SCR is much above the basal error rate, suggesting that these are programmed events with physiological consequences. This is consistent with various evolutionarily conserved functional motifs identified in the C-terminal extensions. We anticipate that more studies will follow to characterize individual SCR events to understand the mechanism of SCR as well as its physiological significance in plants.

## Experimental procedures

### Curation of *A. thaliana* transcriptome

*A. thaliana* has 54,013 transcripts derived from 27,655 protein-coding and 5178 noncoding genes (plants.ensembl.org/Arabidopsis_thaliana/Info/Annotation/#assembly). Sequences of the transcripts were downloaded from Ensembl Plants. From this, we created a file containing sequences of rRNAs, tRNAs, snRNAs, snoRNAs, and miRNAs, which were later used to remove ribosomal footprints that aligned to these sequences. Using cDNA sequences (downloaded from the same source), we noted the positions of the start codon, the canonical stop codon, and the first in-frame stop codon (if any). mRNAs with ISR <45 nucleotides or rest of the 3′UTR <45 nucleotides were removed. This is because sequences with shorter length will not give enough statistical power to draw any conclusions from the ribosomal density differences between them (*i.e.*, ISR and 3′UTR). mRNAs whose ISR sequence was matching with >24 nucleotide sequence of any other CDS were also removed. This was done because reads cannot be mapped onto an ISR if its sequence matches with a CDS. After these filtrations, we were left with 14,732 protein-coding mRNAs for our analysis.

### Preprocessing and sequence alignment of ribosome profiling datasets

SRA-formatted ribosome profiling datasets of nine studies on *A. thaliana* were downloaded from SRA (Table S1). They were converted to FASTQ format files using the prefetch and fastq-dump command of SRAToolkit (https://github.com/ncbi/sra-tools). The adapter sequences were removed (if not removed already) from the datasets using fastp. Additionally, three nucleotides from the 5′ end of all reads were also trimmed using fastp as these nucleotides were generally found to be of a low-quality score. Reads that aligned to noncoding RNA sequences (rRNA, tRNA, snRNA, snoRNA, and miRNA) were removed using Bowtie2 (version: 2.3.4.1). The FASTQ files were then aligned with a list of protein-coding mRNAs to create BAM (Binary alignment map) files.

### Mapping of ribosome profiling (ribo-seq) reads onto the CDS, the ISR, and the 3′UTR of mRNAs

We first analyzed the length distribution of the ribo-seq reads in each dataset. Based on this distribution, we chose the reads of three most abundant lengths for further analyses. Ribo-seq reads were assigned to different regions of an mRNA (*i.e.*, CDS, ISR, and 3′UTR) based on the alignment of the beginning of the read to any of these regions (Fig. S1). To avoid ambiguity during the assignment of the ribo-seq reads to different regions of an mRNA, we followed these criteria:

(i) For CDS: reads that align to the region from 12 nucleotides upstream of the start codon till 22nd nucleotide upstream of the canonical stop codon.
(ii) For ISR: reads that align to the region from 12 nucleotides upstream of the canonical stop codon till 22nd nucleotide upstream of the downstream in-frame stop codon.
(iii) For 3′UTR: reads that align to the region from 12 nucleotides upstream of the downstream in-frame stop codon till the end of the mRNA.

Only those reads that showed 100% alignment to an mRNA region were considered (Even a single mismatch was not allowed).

### Identification of potential SCR candidates

#### Selection based on ribo-seq read density

The mRNAs with higher density of reads in their ISR than that in the CDS were removed from the analysis as this feature is not consistent with SCR. On the contrary, mRNAs with a 4-fold higher density of reads in their ISR than that in the rest of the 3′UTR were included as this is a signature of SCR.

#### Selection based on ribo-seq read coverage

To increase the stringency of this screening process, we applied three more selection criteria based on the coverage:

(i) At least 30 ribo-seq reads should map onto the ISR of a given mRNA.
(ii) >50% of ISR and <25% 3′UTR should be covered by ribo-seq reads. >50% coverage was used as a criterion as ribosome footprints are not found uniformly across a translating region.
(iii) There should be at least one ribo-seq read spanning the canonical stop codon.

#### Selection based on three-nucleotide periodicity

We looked at a 62-nucleotide window length around the start and the stop codons to ensure that the ribo-seq datasets showed three-nucleotide periodicity. To quantify these frame biases, all genes with at least 200 reads in the CDS were considered. Reads were assigned to three frames based on which frame the first nucleotide aligns with an mRNA sequence. We computed the mean and the standard deviation of the fraction of reads that fell in each frame across all the codons of the CDS region. This was then used as the reference distribution against which each of the SCR candidates (filtered based on density and coverage) was compared. The candidates that satisfy the following criterion were selected: the fraction of reads that fell in each frame in the CDS and the ISR regions (test distributions) are within two standard deviations of the reference distribution, in at least one frame.

### Analysis of evolutionary conservation

Homologs of 144 SCR-positive genes of *A. thaliana* were taken from following plant species belonging to Brassicaceae family: *A. lyrata*, *B. rapa*, *C. sativa*, *C. rubella*, *E. salsugineum*, and *R. sativus*. Genes which did not have homologs in at least three species were not included in the analysis. Amino acids sequences potentially encoded by the CDS, ISR, and 3′UTR of homologous genes were aligned using T-coffee multiple sequence alignment tool (Version 13.45.0.4846264), and the alignment score was obtained (50).

### Plasmid constructs

Luciferase constructs for luminescence-based SCR assay were generated in pcDNA 3.1 backbone. The source of all plant cDNAs was *A. thaliana* Col-0 strain. The CDS of the test gene (complete or partial) along with the canonical stop codon and the ISR was cloned upstream of and in-frame with the CDS of firefly luciferase (FLuc) between *Hind*III and *BamH*I sites (*MUB6*, *DXR*, *RPS15AD*, and *ADF1*) or *Kpn*I and *BamH*I sites (*CAM1*, *CURT1B*, and *UPL7*). The FLuc sequence lacked its own start codon. A linker sequence (GGCGGCTCCGGCGGCTCCCTCGTGCTCGGG) was included upstream of the FLuc CDS. Standard PCR-based site-directed mutagenesis was used to generate constructs in which the canonical stop codon of the test genes (TGA in *DXR*, *RPS15AD*, *CAM1*, *CURT1B*, TAG in *MUB6*) was mutated to the other two stop codons. All constructs were verified by sequencing.

For localization studies in HeLa cells, *CURT1B* and *CURT1Bx* (*i.e.*, cDNA of *CURT1B* + its ISR), were cloned between *Kpn*I and *BamH*I sites in pEGFP-C1 vector such that N-terminal GFP-tagged CURT1B isoforms are expressed. In case of *CURT1Bx*, the canonical stop codon TGA was mutated to TCA to maximize its expression. For localization studies in *N. benthamiana* leaves, *CURT1B* and *CURT1Bx* cDNAs with flanking attB sites were generated by PCR. The PCR products were cloned into pDONR221 vector (Invitrogen) using Gateway BP Clonase II Enzyme mix (Thermo Fisher). Next, they were transferred into pGWB652 vector (51) using Gateway LR Clonase II Enzyme Mix (Thermo Fisher), which was then transformed into chemically competent *E. coli* DH5α and then to *Agrobacterium tumefaciens* (GV3101). All constructs were verified by sequencing.

### *In vitro* transcription and translation

The plasmid DNA was linearized using *Not*I enzyme, and 2 μg of the linearized DNA was transcribed *in vitro* using T7 RNA polymerase (Thermo Fisher). The resultant RNA was purified using GeneJET RNA purification kit (Thermo Fisher). The concentration and quality of the RNA were measured using BioPhotometer (Eppendorf). Native agarose gel electrophoresis in TAE or TBE buffer was used to confirm the integrity of the transcribed RNA. The purified RNA (1–3 μg) was *in vitro* translated using wheat germ extract (Promega) at 25 °C for 2 h or using rabbit reticulocyte lysate (Promega) at 30 °C for 2 h as per the manufacturer’s instructions. Luciferase activity was then measured using the Luciferase Assay System (Promega) in the GloMax Explorer System (Promega).

### Nuclear localization studies in Nicotiana leaf

*A. tumefaciens* (GV3101) harboring G3GFP-CURT1B- or G3GFP-CURT1Bx-expressing plasmids were cultured in LB medium containing gentamicin (50 μg/ml), rifampicin (20 μg/ml), and spectinomycin (50 μg/ml) at 28 °C in an incubator shaker. Log-phase culture was subjected to centrifugation at 4 °C and 4000 rpm for 20 min. The pellet was resuspended in 3 ml infiltration solution containing 10 mM MgCl_2_, 10 mM MES-KOH (pH 5.6), and 150 μM acetosyringone and diluted to obtain an absorbance (λ, 600 nm) of 1. This bacterial suspension was infiltrated in the abaxial surface of a *N. benthamiana* leaf using a 1 ml syringe without needle. Two days after infiltration, leaves were cut near the sites of infiltration and washed in ultra-pure water. The leaves were then soaked in aqueous solution of DAPI at 10 μg/ml concentration in room temperature for 20 min. Leaves were mounted on glass slides and imaged using Olympus FLUOVIEW FV3000 confocal microscope (Objective: UPlanSApo 20×/0.75 ∞/0.17/FN26.5). All images were processed and visualized in FV31S-SW FLUOVIEW software.

### Nuclear localization studies in HeLa cells

HeLa cells were cultured in Dulbecco’s Modified Eagle’s Medium (HiMedia) supplemented with 10% fetal bovine serum (Gibco) and 1% antibiotics (10,000 U/ml penicillin, 10,000 μg/ml streptomycin, Lonza). Cells were maintained at 37 °C in a humidified atmosphere with 5% CO_2_. Cells were seeded at 70% to 90% confluency in 6-well plates and transfected with 2 μg of plasmids expressing GFP-tagged CURT1B isoforms using Lipofectamine 2000 (Invitrogen). Twenty-four hours after transfection, the cells were seeded on coverslips for microscopy using Olympus FLUOVIEW FV3000 confocal microscope (Objective: UPlanSApo 60×/1.35 oil ∞/0.17/FN26.5). All images were processed and visualized in FV31S-SW FLUOVIEW software.

## Data availability

All codes used in this study are available at: https://github.com/Divyoj-Singh/Stop_codon_readthrough_pipeline. All data are contained within the manuscript or in the public domain.

## Supporting information

This article contains supporting information.

## Conflict of interest

The authors declare that they have no conflicts of interest with the contents of this article.
