# Supplementary material for: Identification and functional characterization of mRNAs that exhibit stop codon readthrough in Arabidopsis thaliana
Source: J Biol Chem. 2022 Jun 22;298(8):102173. doi: 10.1016/j.jbc.2022.102173 (PMC9293766; doi:10.1016/j.jbc.2022.102173)
Supplement: Legends to Supplementary Figures [file mmc1.docx]

**LEGENDS TO SUPPLEMENTARY FIGURES**

**Figure S1.** Schematic to explain the assignment of ribo-seq reads to various regions of an mRNA.

**Figure S2.** Flow chart showing the four-level screening method to identify mRNAs that show SCR in *A. thaliana*. Blue arrows indicate inclusion and red arrows indicate exclusion.

**Figure S3.** The graphs show fraction of ribo-seq reads in three translation frames on the coding sequences of all protein-coding genes of *A. thaliana*. The data is from the dataset SRP074840 and SRP160376. Red horizontal lines indicate mean values.

**Figure S4.** Evolutionary conservation of ISRs of three genes (*CURT1B*, *KCS12* and *C2H2*) whose SCR products possess putative nuclear localization signal (NLS). The analysis was done using LOCALIZER. Alignment of amino acid sequences (top) and nucleotide sequences (bottom) of the ISR/proximal 3'UTR are shown. Predicted NLSs are highlighted in red. Conservation of nucleotide is indicated with *. Species shown are *Arabidopsis thaliana, Brassica napus, Brassica rapa, Camelina sativa, Eutrema salsugineum*.

**Figure S5.** Evolutionary conservation of ISRs of four genes (*RPL7B, EF1A, ACT2*, and *RGP1*) whose SCR products possess a putative transmembrane helix. Prediction was performed using TMHMM Server v. 2.0. Alignment of amino acid sequences (top) and nucleotide sequences (bottom) of the ISR/proximal 3'UTR are shown. Predicted transmembrane helices are highlighted in red. Conserved nucleotides are indicated with *. Species shown are *Arabidopsis thaliana, Camelina sativa, Capsella rubella and Raphanus sativus.*

**Figure S6.** Evolutionary conservation of ISRs of four genes (*AGP9, At1g07930, DRN1* and *RPL6*) whose SCR products possess a putative transmembrane helix. Prediction was performed using TMHMM Server v. 2.0. Alignment of amino acid sequences (top) and nucleotide sequences (bottom) of the ISR/proximal 3'UTR are shown. Predicted transmembrane helices are highlighted in red. Conserved nucleotides are indicated with *. Species shown are *Arabidopsis thaliana, Eutrema salsugineum, Brassica rapa, Camelina sativa, Capsella rubella and Raphanus sativus.*

**Figure S7.** Evolutionary conservation of ISRs of genes (*IAA2, MAP65, GOX2* and *KCS12*) whose SCR products possess putative intrinsically disordered region (IDR). Alignment of amino acid sequences (top) and nucleotide sequences (bottom) of the ISR/proximal 3'UTR are shown. Predicted IDRs are highlighted in red. Conservation of nucleotide is indicated with *. Species shown are *Arabidopsis thaliana, Capsella rubella, Brassica rapa and Camelina sativa.*

**Figure S8.** Experimental validation of SCR in five *A. thaliana* mRNAs – *DXR, RPS15AD, CURT1B, CAM1* and *MUB6*. Luminescence-based SCR assay. cDNA (full or partial) of a test gene along with the ISR was cloned upstream of and in-frame with the cDNA of firefly luciferase (FLuc) such that FLuc is expressed only if there is SCR across the stop codon of the test cDNA (see the schematic). *ADF1* and *UPL7* were used as negative controls. Constructs were subjected to *in vitro* transcription followed by *in vitro* translation using rabbit reticulocyte lysate with equal amount of RNA. Expression of Fluc was measured by its luminescence activity, which is shown in the graphs. Constructs without ISR were used to measure background signal (first bar), and constructs without any stop codon between the test cDNA and the FLuc were used to measure the maximum luminescence activity (last bar). Statistical significance (two-sided P-value) was obtained using Student’s t-test. Welch’s correction was applied wherever required. Bars show mean ± SD (n=3). Input RNA obtained by *in vitro* transcription is shown below the graphs.
