# Supplementary figures and images for: Identification and functional characterization of mRNAs that exhibit stop codon readthrough in Arabidopsis thaliana
Source: J Biol Chem. 2022 Jun 22;298(8):102173. doi: 10.1016/j.jbc.2022.102173 (PMC9293766; doi:10.1016/j.jbc.2022.102173)

Figure S1

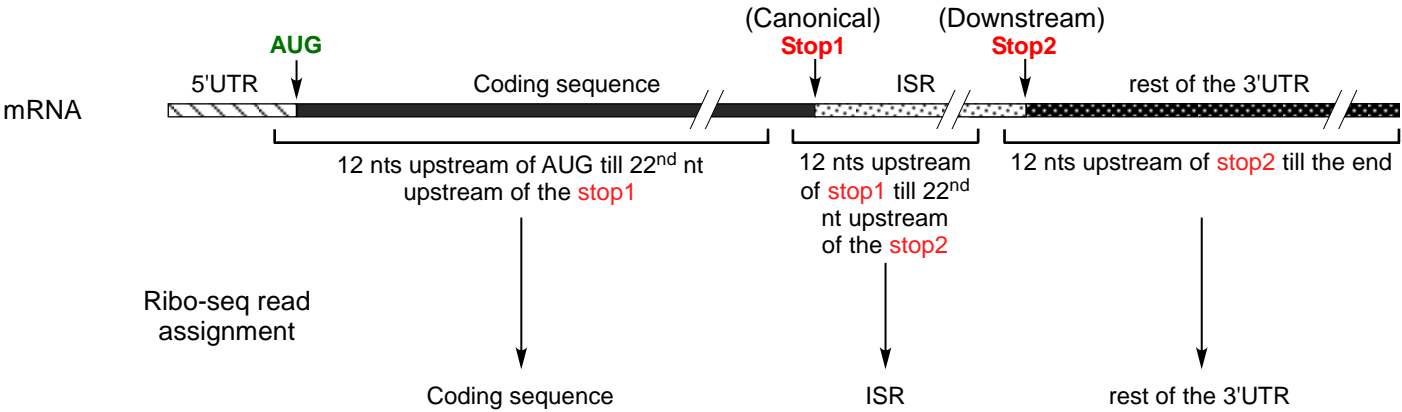

Supplement: Supplemental Figure S1 [file mmc4.pdf]

**Figure S2**

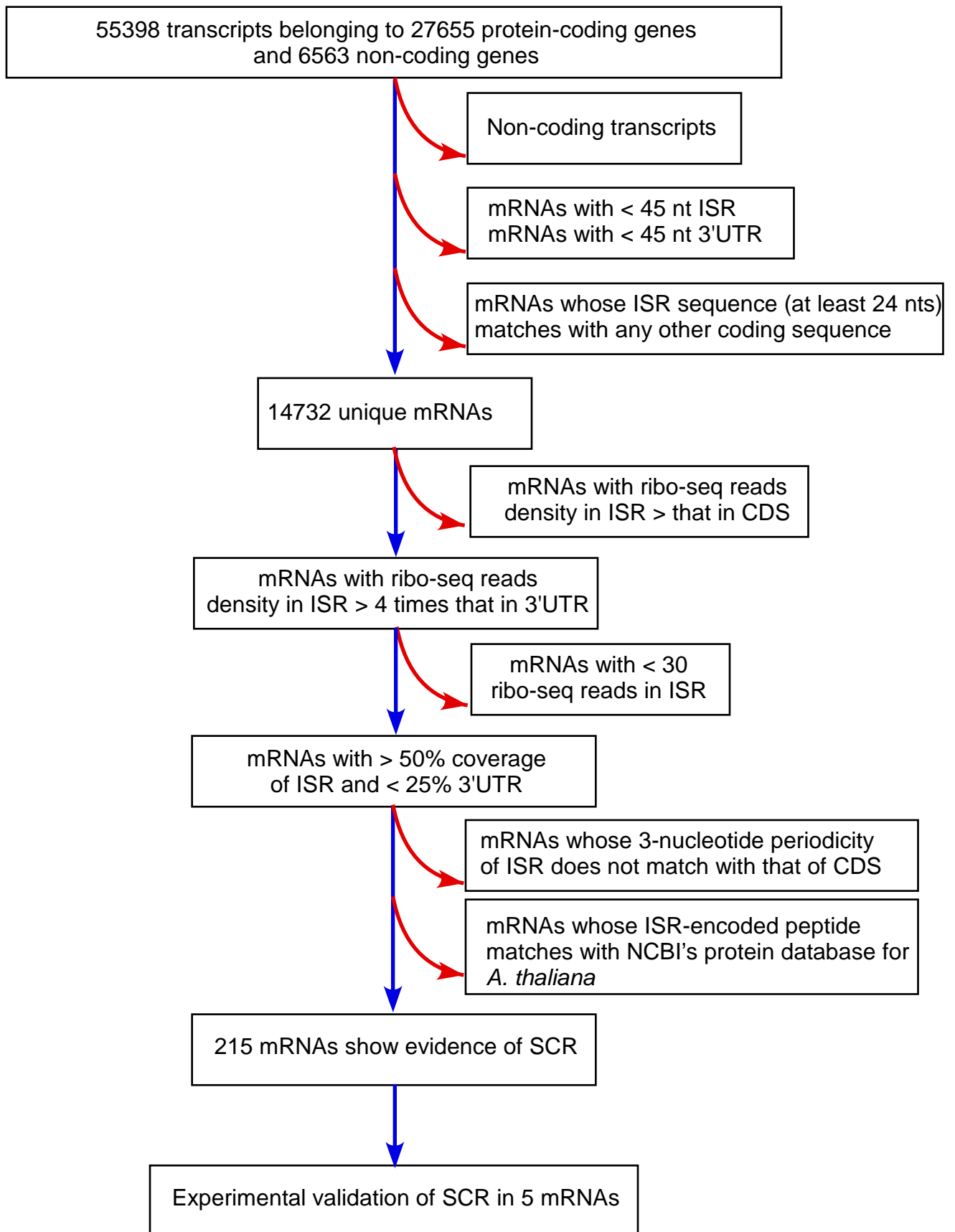

Supplement: Supplemental Figure S2 [file mmc5.pdf]

**Figure S3**

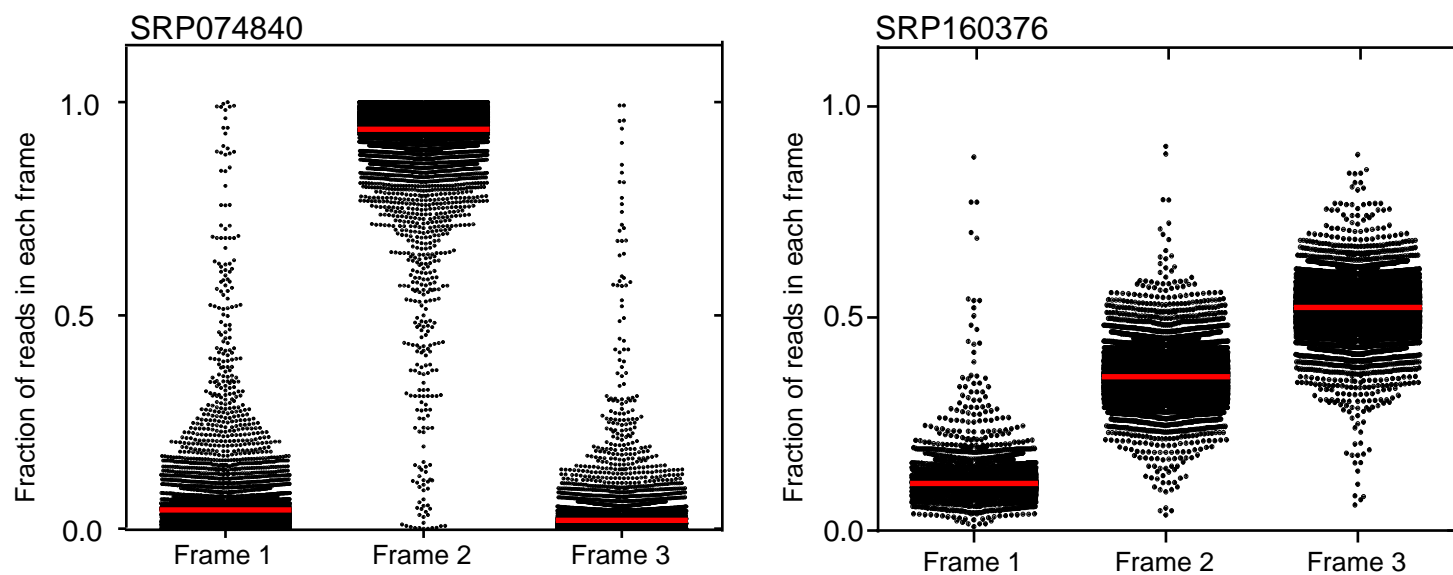

Supplement: Supplemental Figure S3 [file mmc6.pdf]

**Figure S8**

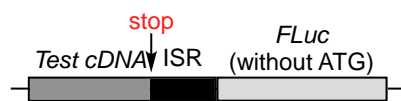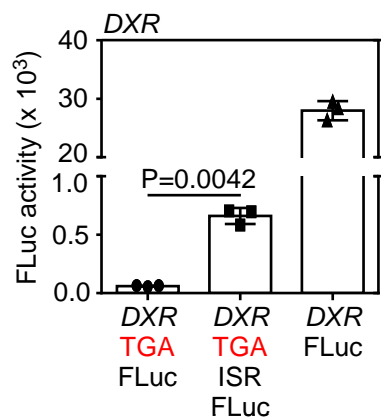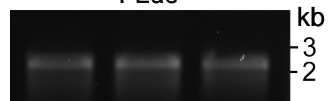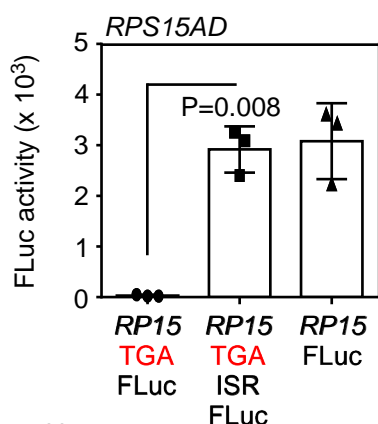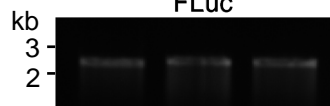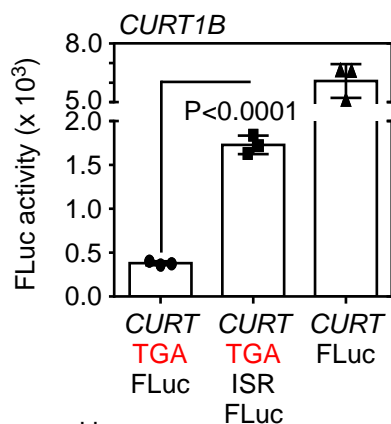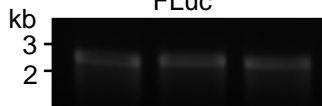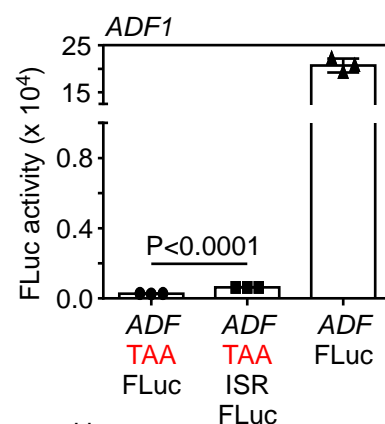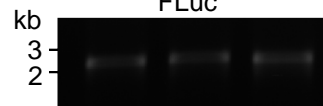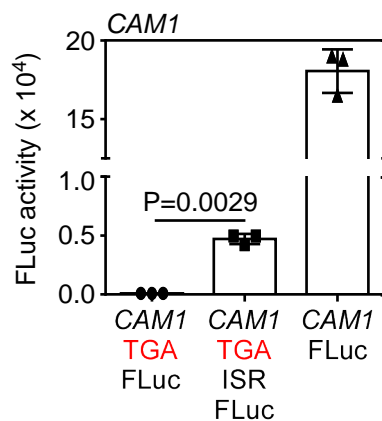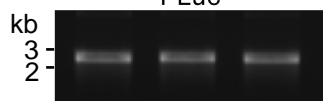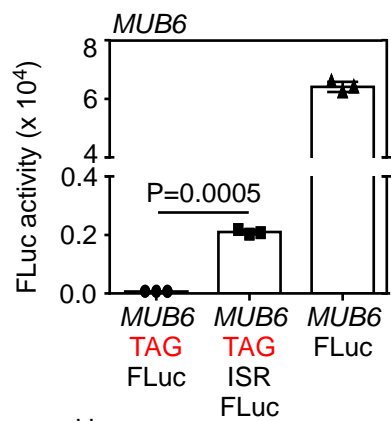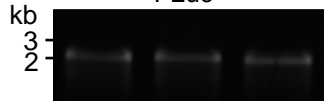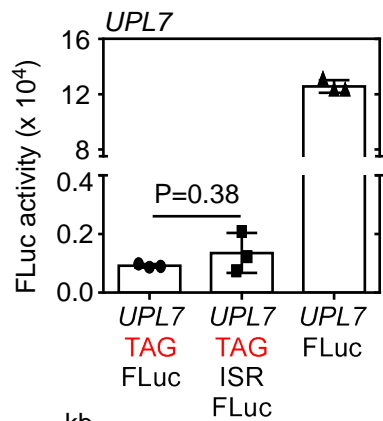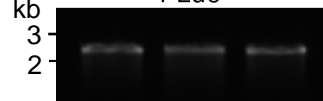

Supplement: Supplemental Figure S8 [file mmc11.pdf]
