# Supplementary material for: Identification and functional characterization of mRNAs that exhibit stop codon readthrough in Arabidopsis thaliana
Source: J Biol Chem. 2022 Jun 22;298(8):102173. doi: 10.1016/j.jbc.2022.102173 (PMC9293766; doi:10.1016/j.jbc.2022.102173)
Supplement: Supplemental Figure S4 [file mmc7.pdf]

Figure S4

CURT1B

```
A. thaliana:     IKGGRRRRRAFLRPFMNWNEGYQKNLTQRPRPSFNLSFL
C. sativa:       IKGGGRRRRALFEAIHEFE
E. salsugineum:  ICGRKRRESRFRPLMNSNEGNQRI

A. thaliana:     ATCAAAGGAGGAAGAAGAAGAAGAGCC-TTTTTGAGGCCATTCATGAATTGGAATGA..
C. sativa:       ATCAAAGGAGGAGGAAGAAGAAGAGCCCTTTTTGAGGCCATTCATGAATTCGAATGA
E. salsugineum:  ATCTGTGGAAGAAAAAGAAGA-GA-GAGCCGTTTT--AGGCCGCTCATGAATTCGAATGA..
                 ***   *** ** ******* ** ***** **** *****  ********* ******
```

KCS12

```
A. thaliana:     NVYAQKRKRKRKNNTRIELVKTCLAIGKPNKCV
C. sativa:       HVYVQKRKRKRKVTQIVLSLS
E. salsugineum:  NVSEQKRKRKNITKVTIFSFFFSFFCFFPPFSVN

A. thaliana:     AACGTTTACGCACAAAAACGCAAACGCAAACG-----CAAAAACAACACAAGGATC..
C. sativa:       CACGTTTACGTACAGAAACGCAAACGCAAACG-----CAAGGTGACACAAATAGTT..
E. salsugineum:  AACGTTTCCGAACAAAAACGCAAACGCAAAAACATTACAAAGGTGACCATATTCTC..
                  ****** ** *** ***************   *   ***              *
```

C2H2 type zinc finger transcription factor family (AT5G56200)

```
A. thaliana:     ETYICKQVIFLTLKKKKTKK
B. napus:        GVQETYICRQVSFFLIFINSKKKVEGKRI
B. rapa:         GVQETYICRQVSFFNFYKPQRKSRRKKNMNLGSISW

A. thaliana:     -GAG--------ACGTATATATGCAAGCAAGTAATCTTTTTAACTTTAAAAAAAAAAAAG..
B. napus:        GGAGTGCAAGAAACGTATATATGCAGACAAGTAAGCTTTTTTTTAATTTTTATAAACTCC..
B. rapa:         GGAGTGCAAGAAACGTATATATGCAGACAAGTAAGCTTTTTTAATTTTTATAAACCCCAA..
                  ***        **************  ******* ******     *    * *
```
