# Supplementary material for: Identification and functional characterization of mRNAs that exhibit stop codon readthrough in Arabidopsis thaliana
Source: J Biol Chem. 2022 Jun 22;298(8):102173. doi: 10.1016/j.jbc.2022.102173 (PMC9293766; doi:10.1016/j.jbc.2022.102173)
Supplement: Supplemental Figure S5 [file mmc8.pdf]

Figure S5

RPL7B

```
A. thaliana:   NVIVFNVKLLCVFIMCLLLDFDLLSQVKRHFESSLLFSLDLNLCFDYCK
C. sativa:     DVFFINVKLLCVFIICLLDFDLLS
C. rubella:    MFSCSMSDFTLFSLYASWILIFFLKLKDFLKLLFICLWFLIWLWTTPGISSLSS

A. thaliana:   AATGTTATCGTTTTCAATGTCAAACTTCTCTGCGTTTTCATTATGTGCCTCCTCCTGGA..
C. sativa:     GATGTTTTCTTTATCAATGTCAAACTTCTCTGTGTTTTCATTATATGCCTCCTTGATTT..
C. rubella:    ATGTTTTCTTGT-TCAATGTCAGACTTCACTCTGTTTTCATTATATGCCTCCTGGATTT..
                   **     * *************** **  *********** ********
```

EF1A

```
A. thaliana:   TLNIKTLSADEMKDYFSFFTLVVWYLVGCLCYRFVYSPSELNSRNWVLDRRWRRYLALELPVYFF..
C. sativa:     TPIIKTLSADESKNVVSLFTLVWYLVACLCYSFVTSPSERCSRNWVLDRRWRSYLAPELFVF
C. rubella:    TPIIKTLSAEESKKYFSFVAVVWCVVACLCYSYSSSPSERCSRNWVLDRRWRSYLAPKLFVFCF

A. thaliana:   ACTCTGAACATCAAAACTCTTTCCGCTGATGAAATGAAGGACTA-TTTTAGTTTCTTTACTTTA..
C. sativa:     ACTCCCATAATCAAAACTCTATCCGCAGATGAA-TCAAAAAATGTTGTTAGTTTGTTTACTCTA..
C. rubella:    ACTCCCATCATCAAAACTCTATCCGCAGAGGAA-TCAAAGAAGTATTTTAGTTTTGTTGCTGTA..
               ****  *  *********** ***** ** *** * **  *    * *******  ** ** **
```

ACT2

```
A. thaliana:   ALKIKGLKSWGFMNGIKVSFFLLYLLLHLFVSFPFLFSFL
C. sativa:     ALQDQRLNKLGFYEWDQKFLFLLSICFSICLFHFLFFFFVFVSMMHLVCDKLSGFYLRLRFKQ..
C. rubella:    ALKIKSWLIKLGFMNGIKSFFFFLFASPFVCFISLFFVFVFHDALVCDKLSGFYLRLRFKTKP..

A. thaliana:   GCTCT-CAAGATCAAA----GGCTTAAAAAGC-TGGGGTTTTATGAATGGGATCAAA-GTTTCT..
C. sativa:     GCTCTTCAAGATCAAA----GGCTTAACAAGCTTGGG-TTTTATGAATGGGATCAAAAGTTTCT..
C. rubella:    GCTCT-CAAGATCAAAAGTTGGCTTATAAAGC-TGGG-TTTTATGAATGGGATCAAAAGTTTCT..
               ***** **********    ******  **** **** ******************* ******
```

RGP1

```
A. thaliana:   AAKKPPPQFWLLAQHIIYLLRFVFVLSFLKFSGDSSVRF
C. rubella:    AAKRPPPQFWLLAQSIIYLLHLVFVFIFSQFSGDSSVRF
R. sativus:    AAKKKKTTTSVLVISSTYIIYLPYLPRFVLSHFPTNSSVSRRSLFK

A. thaliana:   GCAGCAAAAAAA-------CCACCACCGCAGTTTTGGTTATTAGCTCAACATATCATCTATCTT..
C. rubella:    GCAGCAAAAAGA-------CCACCACCACAGTTTTGGTTATTAGCTCAATCTATCATCTATCTT..
R. sativus:    GCAGCAAAAAAAAAAAAAACCACCACCTCAGTTTTGGTTATTAGCTCAACCTAT-ATC-ATCTA..
               ********** *       ******** *********************  ***  ***  ****
```
