# Supplementary material for: Identification and functional characterization of mRNAs that exhibit stop codon readthrough in Arabidopsis thaliana
Source: J Biol Chem. 2022 Jun 22;298(8):102173. doi: 10.1016/j.jbc.2022.102173 (PMC9293766; doi:10.1016/j.jbc.2022.102173)
Supplement: Supplemental Figure S6 [file mmc9.pdf]

Figure S6

AGP9

```
A. thaliana:     VESFVTVLLAFIFMSSLFVPLYCLGLNSFGIYYLSERILLRA
B. rapa:         GGRLFHGVLCNYFYVGRLFVPLLLLWGLYSFGIIIIWRGYY
E. salsugineum:  ARSLSCGVSLHLFLCRLCFVPLLLLWGLNSFGIYYLLERILLRVEMEIRIFCLEIFLWLPFS..

A. thaliana:     GT--GGAAT-CTTTTGTGACTGTGTTAC-TTGCATTTATTTTTATGTCGT-C-TTTGTTT..
B. rapa:         GGG-GGAAGGCTTTTCCA-CGGTGTT-CTTTGCAATTATTTTTATGTCGGACGTTTGTTT..
E. salsugineum:  GCAAGGAGT-CTTTCATG-CGGTGTTTCCTTGCATTTATTTTTATGTCGT-C-TATGTTT..
                 *   ***   ****     * ***** * ***** **************  * * *****
```

(EF-Tu) AT1G07930

```
A. thaliana:     TRILKTLSADESKNNISFFTLVWYLVACYSFVSSPSELCSRNWVLDRRWRSYFAPILLLNCYQF
B. rapa:         TTLRKTLSPAVEWKDKFSFYVTLVLYLVACLCCSFVTSPSELCSRNWVLDRRWRSFSLL
C. rubella:      TPIIKTLSAEESKKYFSFVAVVWCVVACLCYSYSSSPSERCSRNWVLDRRWRSYLAPKLFVFCF

A. thaliana:     ACTCGAATCCTCAAAACTCTAT---CCGCAGATGAATCAAAAAA-CAATATTAGTTTCTTTAC..
B. rapa:         ACTACCTTGAGGAAAACTCTATCACCCGCAGTTGAATGGAAAGA-CAAATTTAGTTTTTATGT..
C. rubella:      ACTCCCATCATCAAAACTCTAT---CCGCAGAGGAATC-AAAGAAGTATTTAGTTTTGTTGC..
                 ***    *    **********   ******  ****  *** *   *  *******   *
```

DRN1

```
A. thaliana:     RFETLKEKNLFGLLCFYMFVSTVIYCFCEKKLCSVTVEFDVMLELVHDRTFVLSILSLM
B. rapa:         RDFLKKSLGYALFCWFLLLSIVFFCL
R. sativus:      RDFEKINLLVVLCSVAFYCYFVCEKNAILQLLLFHIEWVLM

A. thaliana:       AGATTTGAGACTTTAAAAGAGAAAAATCTCTTTGGTT-TGCTATGTTTTTATATGTTTGTTT..
B. rapa:         AGAGATTT-----TTTAAAA-----AAATCTCTT-GGTTATGCTTTGTTCTGTTG-GTTTCTAT..
R. sativus:      AGAGAT-------TTTGAAAAGAT-AAATCTCTT-GGTTGTGCTTTGTTCTGTTG-CTTTCTAT..
                   ****       *** ***     ********* **** **** **** *  *   *** * *
```

RPL6

```
A. thaliana:     SSFFIIIYLLISSFQCLCVFAQICLEMKMFVLGFLSKFLKEPINCF
C. sativa:       SSVSLLCLLTYLLSMLNVYFAHILFLEIEVVCFGILADLFKRNPLIVFDALRNVI
R. sativus:      SSFVPCCCVFYPFYVKCVFAQIFFYEKNVKLFPMIHWLRTY

A. thaliana:     TCTTCA-TTT--TTCATTATTATCTACTTACTTATCTCTTCCTTTCAATGTTTA-TGTGTATTT-GC..
C. sativa:       TCTTCAGTTTCGTTG-TTATG-TCTACTTACTTATCTACTCT---CAATGTTAAATGTGTATTTTGC..
R. sativus:      TCATC--TTTTGTTCCTTGTTGTTGTGTATTTTACCCTTT-----CTATGTTAAATGTGTATTT-GC..
                 ** **  ***  **  ** *  *    *   *** *   *     * ***** * ********* **
```
