# Supplementary material for: Identification and functional characterization of mRNAs that exhibit stop codon readthrough in Arabidopsis thaliana
Source: J Biol Chem. 2022 Jun 22;298(8):102173. doi: 10.1016/j.jbc.2022.102173 (PMC9293766; doi:10.1016/j.jbc.2022.102173)
Supplement: Supplemental Figure S7 [file mmc10.pdf]

Figure S7

IAA2

```
A. thaliana:   SREAENLLSKKEMMTMIDE
C. sativa:     SKEDEKLLLQRR
C. rubella:    SSKRR

A. thaliana:   ---TCCAGA-GAAGCTGAGAATCTTTTGTCTAA--AAAGGAGATGATGACTATGATCGATGAA..
C. sativa:     ---TCCAAA-GAAGATGAGAAGCTTTTGT-T--ACAAAGGAGATGA---CTAGGATCTACGAA..
C. rubella:    TCATCCAAAAGAAGATGAGAAGCTTTTGTTTGCC-AGAGGAGATGA---CTATGATCGATGAA..
                  **** * **** ****** ******* *    * *********   *** **** * ***
```

MAP65

```
A. thaliana:   LDSLFHRICGVMLMVKKEGSEEEGRRLVNTEGD
C. sativa:     LDSLLHRVCGSNVNGKKEGSGRRCRLVNTEGD
C. rubella:    LDSLLHWICGVMLMVKRRCEGDL

A. thaliana:   CTTGACTCTCTTTTCCACCGGATTTGTGGAGTAATGTTAATGGTAAAAAAAGAAGGAAGTGAA..
C. sativa:     CTTGACTCTCTTTTGCACCGGGTTTGTGGGAGTAATGTTAATGGTAAAAAAGAAGGAAGTGGA..
C. rubella:    CTTGACTCTCTTTTGCACTGGATTTGTGGAGTAATGTTAATGGTAAAAAGAAGATGTGAAGGA..
               ****************** ** *******    *   * *  *  **** *  * *    * *
```

GOX2

```
A. thaliana:   RRKKKQRTETTRHQNVFIF
C. sativa:     RKKKKNNNTEHVMPKPIHILIDHMAFYSFFFFF
C. rubella:    RKKKNKKKTTTTQSM

A. thaliana:   AGAAGAAAAAAAAA--ACAGAGAACAGAAACAACACGGCACCAAAACGTATTCATATTCTGA..
C. sativa:     AGAAAAAAAAAAAAAAACAACAACACAGAGCATGTAATGCCTAAACCTATTCATATTCTGAT..
C. rubella:    AGAAAAAAAAAAAA---CAAAAAAAAAACAACAACAACACAGAGCATGTAATGCCTAAACCT..
               **************   **   *                   *
```

KCS12

```
A. thaliana:   NVYAQKRKRKRKNNTRIELVKTCLAIGKPNKCV
C. sativa:     HVYVQKRKRKRKVTQIVLSLS
B. rapa:       NVSAQKRKRENNTKGDNIFFFFFFFFFPFPLS

A. thaliana    AACGTTTACGCACAAAAACGCAAACGCAAACGCAAAAACAACACAAGGATCGAATTAGTT..
C. sativa      CACGTTTACGTACAGAAACGCAAACGCAAACGCAAGGTGACACAAATAGTTCTCTCTCTC..
B. rapa        AACGTTTCCGCACAAAAACGCAAACGCGAAAATAACACAAAAGGTGACAATATTTTCTTC..
                ****** ** *** ************ **   **    *              *   *
```
